# Association of preoperative D-dimer-to-albumin ratio and prognostic nutritional index with aseptic wound complications and periprosthetic joint infection after primary total knee arthroplasty: a single-center retrospective cohort study

**DOI:** 10.1186/s12891-026-10158-8

**Published:** 2026-07-10

**Authors:** Zhi-Chao Han, Xiao-Kai Liu, Peng-Yuan Su, Yuan-He Wang, Shuo Yuan, Bin-Hai Yu, Zhen Liu, Hong-Yu Wang, Yan Wang, Shao-Qi Tian

**Affiliations:** 1 Department of Orthopedic Surgery, The Affiliated Hospital of Qingdao University, 16 Jiangsu Road, Qingdao, 266071 Shandong China; 2 Department of Gynecology, The Women and Children’s Hospital of Qingdao, 16 Jiangsu Road, Qingdao, 266071 Shandong China

**Keywords:** Total knee arthroplasty, Periprosthetic joint infection, Aseptic wound complications, D-dimer-to-albumin ratio, Prognostic nutritional index, Risk assessment

## Abstract

**Objective:**

To investigate the associations of the preoperative D-dimer-to-albumin ratio (DAR) and prognostic nutritional index (PNI) with aseptic wound complications (AWC) and periprosthetic joint infection (PJI) after primary total knee arthroplasty (TKA), and to evaluate their within-sample discrimination for distinct postoperative adverse outcomes.

**Methods:**

This single-center retrospective cohort study included patients who underwent primary unilateral TKA at the Affiliated Hospital of Qingdao University between April 2019 and January 2024. Patients with both AWC and PJI were classified in the PJI group only. Deep venous thrombosis was excluded preoperatively using lower-extremity color Doppler ultrasonography. DAR and PNI were calculated from laboratory tests obtained within 48 h before surgery. AWC was assessed within 30 days after surgery, and PJI was assessed within 2 years according to the 2018 International Consensus Meeting criteria. Multivariable logistic regression, receiver operating characteristic (ROC) analysis with 95% confidence intervals (CIs), DeLong tests, bootstrap internal validation, calibration assessment, and sensitivity analyses were performed.

**Results:**

A total of 1,197 patients were included, including 45 isolated AWC events and 35 PJI events. After adjustment for age, sex, body mass index, diabetes mellitus, and Charlson Comorbidity Index, preoperative PNI was independently associated with isolated AWC (odds ratio [OR], 0.613; 95% CI, 0.543–0.693; *P* < 0.001), whereas preoperative DAR was not associated with isolated AWC (OR, 0.952; 95% CI, 0.852–1.064; *P* = 0.388). In contrast, DAR was independently associated with PJI (OR, 1.454; 95% CI, 1.328–1.592; *P* < 0.001), whereas PNI was not significantly associated with PJI (OR, 0.915; 95% CI, 0.825–1.015; *P* = 0.094). PNI showed an AUC of 0.873 (95% CI, 0.792–0.955) for isolated AWC, and DAR showed an AUC of 0.920 (95% CI, 0.865–0.975) for PJI. Bootstrap optimism-corrected AUCs were 0.862 for the adjusted PNI-AWC model and 0.900 for the adjusted DAR-PJI model.

**Conclusions:**

In this exploratory single-center retrospective cohort, preoperative PNI was more strongly associated with isolated AWC, whereas preoperative DAR was more strongly associated with PJI. Although both markers showed good apparent discrimination and high negative predictive values, the low event numbers, limited positive predictive values, imperfect calibration, and absence of external validation require cautious interpretation. Larger multicenter prospective studies are needed before these cutoffs can be applied as clinical decision thresholds.

## Introduction

In total knee arthroplasty (TKA), aseptic wound complications (AWC) and periprosthetic joint infection (PJI) are among the most clinically relevant postoperative complications. AWC typically occurs in the early postoperative period and may interfere with incision healing, prolong dressing changes and hospitalization, and potentially be associated with subsequent infectious events [1]. PJI, by contrast, is one of the most devastating complications after joint arthroplasty and is associated with complex treatment pathways, repeated operations, prolonged antimicrobial therapy, functional impairment, increased mortality risk, and substantial health-economic burden [2–5]. Therefore, preoperative identification of patients at increased risk of adverse outcomes remains important for individualized perioperative management.

Nutritional-immune status and coagulation-inflammatory abnormalities have increasingly been recognized as clinically relevant dimensions of perioperative risk. The prognostic nutritional index (PNI), which incorporates serum albumin and total lymphocyte count (TLC), provides a convenient composite measure of nutritional reserve and immune status. In arthroplasty populations, low PNI, hypoalbuminemia, or broader malnutrition has been associated with wound complications, infection, and reintervention risk [6–12]. Meanwhile, D-dimer reflects fibrinolytic activation and coagulation response, whereas albumin is closely related to inflammatory burden, capillary permeability, and nutritional status. The D-dimer-to-albumin ratio (DAR), as a composite marker, has recently been used in orthopedic thrombotic risk assessment and infectious or critical illness settings to capture hypercoagulability together with systemic inflammatory-nutritional imbalance [13, 14].

Many previous studies have combined total hip and total knee arthroplasty, or have focused primarily on PJI diagnosis and infection-related biomarkers, with limited differentiation between AWC and PJI. These two outcomes may be related clinically but are not identical in timing, biological basis, or downstream implications [7–9, 15–18]. Accordingly, this study aimed to investigate the associations of preoperative DAR and PNI with isolated AWC and PJI after primary unilateral TKA, and to evaluate the discriminative performance and internal robustness of these markers while using a cautious exploratory interpretation.

## Materials and methods

### Study design and ethics

This was a single-center retrospective cohort study. Patients who underwent primary unilateral TKA at the Affiliated Hospital of Qingdao University between April 2019 and January 2024 were screened for inclusion. The study protocol conformed to the Declaration of Helsinki and was approved by the Ethics Committee of the Affiliated Hospital of Qingdao University (approval No. 42158 QYFY WZLL). According to institutional policy, all patients had provided written informed consent for the use of their clinical data for research purposes. The primary outcomes were postoperative isolated AWC and PJI. PJI was defined according to the 2018 International Consensus Meeting (ICM) criteria [19].

### Study population

**Fig. 1:**
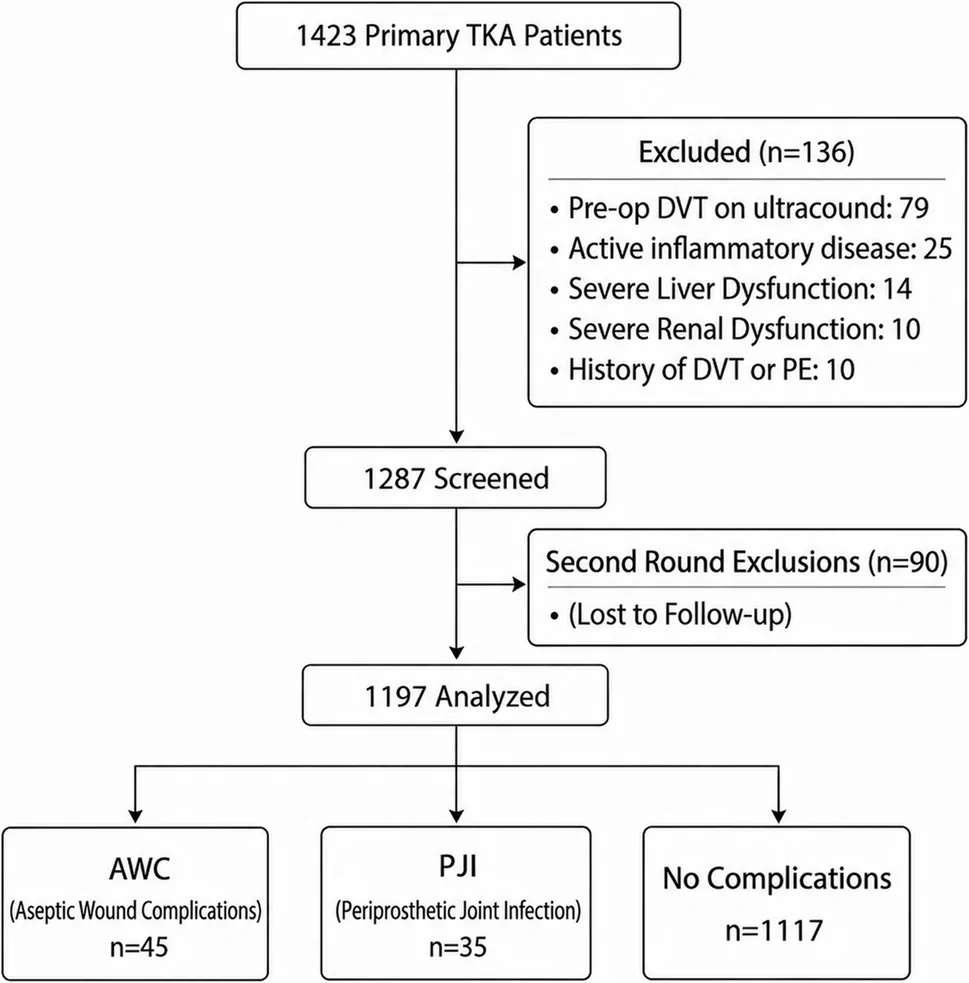
Flowchart of patient selection. Patients with both AWC and PJI were classified as PJI only. Abbreviations: TKA, total knee arthroplasty; AWC, aseptic wound complications; PJI, periprosthetic joint infection; DVT, deep venous thrombosis; PE, pulmonary embolism

Inclusion criteria were as follows: (1) primary knee osteoarthritis; (2) primary unilateral TKA; and (3) complete laboratory data within 48 h before surgery for DAR and PNI calculation. Exclusion criteria were: (1) thrombosis detected on preoperative lower-extremity vascular ultrasonography; (2) a history of venous thrombosis or pulmonary embolism; (3) active malignancy or recent chemotherapy/radiotherapy; (4) inflammatory arthropathy; (5) severe hepatic dysfunction or nephrotic syndrome; (6) major trauma or surgery within the previous 3 months; and (7) missing key clinical data. Patients with both AWC and PJI were classified in the PJI group only. The patient-selection flowchart is shown in Fig. 1.

### Data collection and variable calculation

Demographic characteristics, comorbidities, perioperative variables, and laboratory data were extracted from the electronic medical record system. Variables included age, sex, body mass index (BMI), operative side, diabetes mellitus, hypertension, coronary artery disease, smoking status, Charlson Comorbidity Index (CCI), American Society of Anesthesiologists (ASA) classification, operative time, estimated blood loss, drainage volume, and available inflammatory markers including white blood cell count (WBC), C-reactive protein (CRP), and erythrocyte sedimentation rate (ESR).

Preoperative laboratory tests used for DAR and PNI calculation, including D-dimer, serum albumin, and TLC, were obtained within 48 h before surgery. Chest computed tomography (CT) was not performed for research purposes or specifically for TKA surgical planning. According to our institutional preoperative anesthetic and pulmonary risk assessment pathway, patients aged 60 years or older undergoing elective TKA generally underwent chest CT after being informed of potential radiation exposure and after providing consent. For patients who had undergone chest CT within the preceding 6 months and whose clinical condition had not substantially changed, the existing CT results were accepted and repeat CT was not performed. Therefore, all included patients had available chest CT information within 6 months before surgery or during the index admission. This institutional practice was considered part of routine clinical care rather than a study-mandated examination [20–22].

DAR and PNI were independently calculated and cross-checked by two investigators; discrepancies were adjudicated by a third investigator. DAR = D-dimer (ng/mL)/albumin (g/L); PNI = albumin (g/L) + 5 × TLC (×10^9/L).

### Surgical procedure and perioperative management

All procedures were performed by the same senior arthroplasty team in a laminar-flow operating room. Spinal anesthesia combined with epidural anesthesia was preferred, with conversion to general anesthesia when necessary. A tourniquet (280 mmHg) was applied intraoperatively, and a standard anterior midline incision with a medial parapatellar approach was used, followed by conventional bone resection, soft-tissue balancing, and cemented fixation. One drainage tube was routinely placed and removed at 24 ± 2 h postoperatively. Intra-articular tranexamic acid (1 g) was administered during surgery.

Prophylactic antibiotics consisted of intravenous cefazolin 2 g, administered as two 1-g vials, 30 min before surgery and repeated every 12 h for 48 h postoperatively according to our institutional perioperative protocol [23]. Venous thromboembolism prophylaxis included low-molecular-weight heparin during hospitalization and rivaroxaban 10 mg/day after discharge for 35 days. In-hospital anticoagulation was administered under medical supervision. After discharge, medication education was reinforced during dressing changes, suture removal, and outpatient follow-up visits; however, objective post-discharge adherence data, such as pill counts or pharmacy refill records, were not available in this retrospective database. Dressings were routinely changed on postoperative days 3–5, sutures were removed on postoperative day 14, and outpatient follow-up visits were scheduled at 30 and 90 days after surgery.

### Outcome definitions and follow-up

AWC was defined as a noninfectious event related to abnormal local wound healing within 30 days after surgery. Patients were required to have no clear signs of purulent infection and not meet the criteria for surgical site infection or the 2018 PJI definition. AWC was treated as a composite endpoint comprising any of the following: (1) persistent wound drainage, defined as ongoing drainage beyond 72 h postoperatively; (2) superficial wound dehiscence involving only the skin/subcutaneous layer without deep fascial disruption; (3) hematoma/seroma, defined as localized bloody or serous fluid collection around the incision without purulent features; (4) skin-edge necrosis, defined as partial- or full-thickness skin devitalization/eschar at the wound edge; and (5) delayed healing, defined as failure to achieve a dry closed wound by postoperative day 14 or the need for continued dressing changes, delayed suture removal, or other local wound care due to poor healing. For patients with wound problems, joint aspiration was performed under aseptic conditions according to the institutional workflow when clinically indicated, and synovial fluid white blood cell count, neutrophil percentage, and microbiological culture were assessed to exclude infection. Patients with both AWC and PJI were classified as PJI only, and the AWC group therefore represented isolated AWC.

PJI was diagnosed according to the 2018 ICM criteria and assessed within 2 years after surgery [19]. Outcomes within 30 days postoperatively were primarily obtained from inpatient and outpatient medical records. Long-term PJI surveillance was maintained through outpatient follow-up records, readmission records, and telephone follow-up. Immediate family members were used as backup contacts when patients could not be reached directly. When patients reported diagnosis or treatment outside our institution, available external medical documents were reviewed when possible. All patients had at least 2 years of outcome data available, and follow-up was censored in February 2026.

For descriptive analysis, PJI cases were further categorized according to timing and presumed clinical route. Acute postoperative PJI was defined as PJI diagnosed within 90 days after the index TKA. Delayed-period PJI was defined by timing as PJI diagnosed between 91 days and 24 months after surgery. Among delayed-period cases, those without evidence suggesting hematogenous seeding were classified as delayed non-hematogenous PJI, whereas cases with a distant infectious focus and/or organism-source evidence compatible with hematogenous seeding were classified as presumed hematogenous PJI. When the available clinical information was insufficient to determine the presumed route of infection, the case was considered unclassifiable with respect to infection route. This classification was used for descriptive purposes only and did not replace the 2018 ICM diagnostic criteria.

### Statistical analysis

Statistical analyses were performed using SPSS version 25.0 (IBM Corp., Armonk, NY, USA) and R/Python-based statistical routines for ROC comparison, bootstrap validation, calibration, and decision-curve analyses. Data completeness and logical consistency were checked before formal analysis. Patients with missing values in variables required for a specific model were excluded from that model; no imputation was performed. This complete-case approach was used because the primary predictors and main covariates had nearly complete availability, whereas CRP and ESR were used mainly in supplemental sensitivity analyses.

Continuous variables were assessed for normality using the Shapiro-Wilk test and inspection of distributional plots. Because several continuous variables, especially D-dimer and DAR, deviated from normality, continuous variables are reported as both mean ± standard deviation and median (interquartile range), and group comparisons primarily used the Mann-Whitney U test. Welch’s t test results were retained as sensitivity analyses for comparability with the original analysis. Categorical variables are presented as number (percentage) and were compared using the chi-square test or Fisher’s exact test, as appropriate.

Multivariable logistic regression models were constructed separately for isolated AWC and PJI to examine the associations of DAR and PNI with postoperative adverse outcomes after adjustment for prespecified clinical variables: age, sex, BMI, diabetes mellitus, and CCI. Because DAR is mathematically related to D-dimer and albumin and PNI is mathematically related to albumin and TLC, composite-marker models and component-marker models were analyzed separately to avoid obvious collinearity. This model separation was prespecified as a methodologic safeguard rather than a data-driven selection strategy.

Given the limited number of outcome events, particularly PJI events, the primary multivariable models were kept parsimonious. Events-per-variable (EPV) was calculated to evaluate overfitting risk. Surgery-related variables (operative time, estimated blood loss, and drainage volume), smoking status, and standard inflammatory markers (WBC, CRP, and ESR) were evaluated in exploratory sensitivity analyses rather than incorporated into the primary models. Incremental value beyond clinical variables and standard laboratory markers was assessed by comparing nested models using changes in AUC, likelihood-ratio testing, Akaike information criterion, and Bayesian information criterion.

ROC curve analysis was performed to assess the discriminative performance of DAR, PNI, their component markers, and standard inflammatory markers for isolated AWC and PJI. AUCs with 95% confidence intervals were calculated, and DeLong tests were used for formal comparison between correlated AUCs. Optimal cutoffs were identified using the Youden index and were interpreted as exploratory thresholds. Bootstrap resampling with 1,000 iterations was used for internal validation and estimation of optimism-corrected AUCs. Calibration was assessed using Brier score and the Hosmer-Lemeshow test, and decision-curve analysis was performed to estimate net benefit across selected threshold probabilities. All tests were two-sided, and *P* < 0.05 was considered statistically significant.

## Results

### Baseline characteristics and event occurrence

A total of 1,197 patients who underwent primary unilateral TKA were included. According to the prespecified grouping rule, patients with both AWC and PJI were classified as PJI only; thus, 45 patients had isolated AWC, 35 patients had PJI, and 1,117 patients had neither AWC nor PJI. In the overall cohort, the mean age was 70.1 ± 6.93 years and the mean BMI was 26.6 ± 3.46 kg/m²; 816 patients (68.2%) were women and 248 (20.7%) had diabetes mellitus. The median preoperative D-dimer level was 370 ng/mL (IQR, 300–460), the mean albumin level was 44.9 ± 2.56 g/L, the mean PNI was 54.2 ± 3.42, and the mean DAR was 8.7 ± 2.86 (Table 1).

**Table 1:** Baseline characteristics of the overall study cohort

Characteristic	Available *n*	Mean ± SD or *n* (%)	Median (IQR)
Age, years	1197	70.06 ± 6.93	70.00 (66.00–74.00)
BMI, kg/m²	1197	26.57 ± 3.46	26.12 (23.94–28.78)
D-dimer, ng/mL	1197	388.14 ± 123.09	370.00 (300.00-460.00)
Albumin, g/L	1197	44.85 ± 2.56	44.80 (43.20–46.60)
TLC, ×10^9/L	1197	1.86 ± 0.53	1.82 (1.52–2.09)
WBC, ×10^9/L	1197	6.25 ± 1.51	6.24 (5.17–7.29)
CRP, mg/L	1194	1.41 ± 2.17	0.25 (0.25–1.98)
ESR, mm/h	1196	10.07 ± 5.22	9.00 (6.00–14.00)
PNI	1197	54.17 ± 3.42	54.20 (51.90-56.35)
DAR	1197	8.69 ± 2.86	8.19 (6.65–10.28)
Operative time, min	1197	89.50 ± 5.24	90.00 (85.00–95.00)
Estimated blood loss, mL	1197	20.13 ± 3.55	20.00 (16.00–23.00)
Drainage volume, mL	1197	173.60 ± 47.38	190.00 (160.00-200.00)
Female sex	1197	816 (68.2%)	
Diabetes mellitus	1197	248 (20.7%)	
Hypertension	1197	238 (19.9%)	
Coronary artery disease	1197	132 (11.0%)	
Current/former smoker	1197	236 (19.7%)	
Right-side surgery	1197	625 (52.2%)	
ASA I/II/III	1196	27 (2.3%)/1027 (85.9%)/142 (11.9%)	
CCI low/intermediate/high risk	1197	762 (63.7%)/370 (30.9%)/65 (5.4%)	

Data are presented as mean ± standard deviation with median (interquartile range) for continuous variables, or n (%) for categorical variables. Abbreviations: *BMI *body mass index, *TLC *total lymphocyte count, *WBC *white blood cell count, *CRP *C-reactive protein, *ESR *erythrocyte sedimentation rate, *PNI *prognostic nutritional index, *DAR *D-dimer-to-albumin ratio, *ASA *American Society of Anesthesiologists, *CCI *Charlson Comorbidity Index

### Baseline comparison between the isolated AWC and non-AWC groups

After stratification by isolated AWC, 45 patients were classified into the AWC group and 1,152 into the non-AWC group. The Shapiro-Wilk test showed that D-dimer significantly deviated from normality in the overall cohort (W = 0.944, *P* < 0.001), and natural-log transformation did not resolve the distributional departure (W = 0.927, *P* < 0.001). Therefore, Mann-Whitney U tests were used as the primary group-comparison method for D-dimer and other non-normally distributed continuous variables. D-dimer did not differ significantly between the isolated AWC and non-AWC groups [350 (190–470) vs. 370 (300–460) ng/mL; *P* = 0.140]. Compared with the non-AWC group, the isolated AWC group had lower preoperative albumin levels and lower PNI values. TLC and CRP showed small distributional differences by non-parametric testing, whereas ESR, operative time, estimated blood loss, drainage volume, and smoking status did not differ significantly between groups (Table 2).

**Table 2:** Baseline characteristics of the isolated AWC and non-AWC groups

Characteristic	Isolated AWC (*N* = 45)	Non-AWC (*N* = 1152)	*P* value
Age, years	70.04 ± 5.12; 69.00 (67.00–75.00)	70.07 ± 6.99; 70.00 (66.00–74.00)	0.892
BMI, kg/m²	26.07 ± 3.21; 25.89 (23.53–28.01)	26.59 ± 3.46; 26.12 (24.03–28.89)	0.404
D-dimer, ng/mL	336.67 ± 177.96; 350.00 (190.00-470.00)	390.15 ± 120.10; 370.00 (300.00-460.00)	0.140
Albumin, g/L	41.07 ± 3.36; 41.10 (39.40–43.00)	45.00 ± 2.41; 45.00 (43.30–46.60)	< 0.001
TLC, ×10^9/L	1.77 ± 0.68; 1.59 (1.27–2.01)	1.87 ± 0.52; 1.82 (1.53–2.09)	0.033
WBC, ×10^9/L	6.10 ± 1.53; 5.95 (5.03–7.34)	6.26 ± 1.51; 6.31 (5.17–7.29)	0.434
CRP, mg/L	1.70 ± 1.46; 1.86 (0.25–2.98)	1.39 ± 2.19; 0.25 (0.25–1.91)	0.035
ESR, mm/h	10.27 ± 4.36; 9.00 (7.00–14.00)	10.07 ± 5.25; 9.00 (6.00–14.00)	0.501
PNI	49.85 ± 4.02; 48.95 (48.15–49.35)	54.34 ± 3.28; 54.30 (52.10-56.35)	< 0.001
DAR	8.32 ± 4.53; 9.49 (4.32–11.40)	8.71 ± 2.78; 8.18 (6.65–10.26)	0.814
Operative time, min	89.56 ± 5.20; 90.00 (85.00–95.00)	89.50 ± 5.24; 90.00 (85.00–95.00)	0.962
Estimated blood loss, mL	20.20 ± 3.74; 20.00 (16.00–24.00)	20.13 ± 3.55; 20.00 (16.00–23.00)	0.888
Drainage volume, mL	173.11 ± 46.26; 180.00 (160.00-200.00)	173.62 ± 47.44; 190.00 (160.00-200.00)	0.751
Female sex	30 (66.7%)	786 (68.2%)	0.825
Diabetes mellitus	9 (20.0%)	239 (20.7%)	0.904
Hypertension	7 (15.6%)	231 (20.1%)	0.458
Coronary artery disease	5 (11.1%)	127 (11.0%)	1.000
Right-side surgery	20 (44.4%)	605 (52.5%)	0.288
ASA I/II/III	1/40/4	26/987/138	0.818
CCI low/intermediate/high	27/15/3	735/355/62	0.853
Current/former smoker	11 (24.4%)	225 (19.5%)	0.416

Continuous variables are presented as mean ± standard deviation; median (interquartile range). Primary P values for continuous variables were calculated using the Mann-Whitney U test. Categorical variables were compared using the chi-square test or Fisher exact test. Patients with both AWC and PJI were classified in the PJI group only; therefore, the AWC group represents isolated AWC

### Baseline comparison between the PJI and non-PJI groups

After stratification by PJI, 35 patients were classified into the PJI group and 1,162 into the non-PJI group. Compared with the non-PJI group, the PJI group had markedly higher preoperative D-dimer levels [570 (520–650) vs. 360 (300–450) ng/mL; *P* < 0.001], higher DAR values, and lower albumin levels. No significant group differences were observed for age, BMI, TLC, PNI, WBC, CRP, ESR, operative time, estimated blood loss, drainage volume, smoking status, diabetes mellitus, or CCI (Table 3).

**Table 3:** Baseline characteristics of the PJI and non-PJI groups

Characteristic	PJI (*N* = 35)	Non-PJI (*N* = 1162)	*P* value
Age, years	69.63 ± 8.68; 69.00 (64.50–75.00)	70.08 ± 6.88; 70.00 (66.00–74.00)	0.976
BMI, kg/m²	26.87 ± 3.34; 27.04 (23.69–28.26)	26.56 ± 3.46; 26.12 (24.03–28.78)	0.640
D-dimer, ng/mL	594.00 ± 166.68; 570.00 (520.00-650.00)	381.94 ± 116.09; 360.00 (300.00-450.00)	< 0.001
Albumin, g/L	43.29 ± 3.46; 42.90 (41.35–45.80)	44.90 ± 2.51; 44.85 (43.20–46.60)	0.002
TLC, ×10^9/L	1.99 ± 0.66; 1.87 (1.61–2.08)	1.86 ± 0.53; 1.82 (1.52–2.09)	0.448
WBC, ×10^9/L	6.04 ± 1.40; 5.79 (4.89–6.87)	6.26 ± 1.51; 6.25 (5.17–7.29)	0.336
CRP, mg/L	1.38 ± 1.36; 0.78 (0.25–2.45)	1.41 ± 2.19; 0.25 (0.25–1.97)	0.315
ESR, mm/h	10.91 ± 5.88; 10.00 (6.00-15.50)	10.05 ± 5.20; 9.00 (6.00–14.00)	0.410
PNI	53.22 ± 3.63; 53.00 (51.73–55.45)	54.20 ± 3.41; 54.20 (51.90-56.35)	0.212
DAR	13.78 ± 3.76; 12.80 (12.15–14.99)	8.54 ± 2.68; 8.13 (6.64–9.95)	< 0.001
Operative time, min	89.14 ± 5.35; 90.00 (85.00–95.00)	89.51 ± 5.24; 90.00 (85.00–95.00)	0.680
Estimated blood loss, mL	20.31 ± 3.36; 20.00 (18.00–23.00)	20.13 ± 3.56; 20.00 (16.00-23.75)	0.743
Drainage volume, mL	172.57 ± 46.85; 190.00 (160.00-200.00)	173.63 ± 47.42; 190.00 (160.00-200.00)	0.841
Female sex	24 (68.6%)	792 (68.2%)	0.959
Diabetes mellitus	9 (25.7%)	239 (20.6%)	0.459
Hypertension	9 (25.7%)	229 (19.7%)	0.380
Coronary artery disease	4 (11.4%)	128 (11.0%)	0.789
Right-side surgery	17 (48.6%)	608 (52.3%)	0.661
ASA I/II/III	1/29/5	26/998/137	0.873
CCI low/intermediate/high	22/11/2	740/359/63	0.994
Current/former smoker	10 (28.6%)	226 (19.4%)	0.181

Continuous variables are presented as mean ± standard deviation; median (interquartile range). Primary P values for continuous variables were calculated using the Mann-Whitney U test. Patients with isolated AWC are included in the non-PJI group

### Multivariable logistic regression analyses

After adjustment for age, sex, BMI, diabetes mellitus, and CCI, the isolated AWC regression model showed no significant association between preoperative DAR and isolated AWC (OR, 0.952; 95% CI, 0.852–1.064; *P* = 0.388), whereas preoperative PNI was significantly associated with isolated AWC (OR, 0.613; 95% CI, 0.543–0.693; *P* < 0.001). In contrast, in the PJI regression model, preoperative DAR was significantly associated with PJI (OR, 1.454; 95% CI, 1.328–1.592; *P* < 0.001), whereas PNI was not significantly associated with PJI (OR, 0.915; 95% CI, 0.825–1.015; *P* = 0.094) (Table 4).

**Table 4:** Main multivariable logistic regression analyses for isolated AWC and PJI

Outcome/model	Marker	Adjusted OR (95% CI)	*P* value	Covariates
Isolated AWC: DAR model	DAR	0.952 (0.852–1.064)	0.388	Age, sex, BMI, diabetes, CCI
Isolated AWC: PNI model	PNI	0.613 (0.543–0.693)	< 0.001	Age, sex, BMI, diabetes, CCI
PJI: DAR model	DAR	1.454 (1.328–1.592)	< 0.001	Age, sex, BMI, diabetes, CCI
PJI: PNI model	PNI	0.915 (0.825–1.015)	0.094	Age, sex, BMI, diabetes, CCI

ORs are per 1-unit increase in each marker. Composite-marker models were analyzed separately from component-marker models to reduce collinearity. Abbreviations: *OR *odds ratio, *CI *confidence interval, *BMI *body mass index, *CCI *Charlson Comorbidity Index

### ROC analyses with confidence intervals and formal marker comparisons

ROC analysis showed that PNI had the highest AUC for isolated AWC among the evaluated markers (AUC, 0.873; 95% CI, 0.792–0.955), with an exploratory cutoff of 49.95 (sensitivity, 86.7%; specificity, 93.2%; PPV, 33.3%; NPV, 99.4%). Albumin also showed good discrimination for isolated AWC (AUC, 0.844; 95% CI, 0.779–0.908). DeLong testing showed that the AUC of PNI was not statistically different from that of albumin (*P* = 0.293), although PNI was significantly higher than TLC and DAR (both *P* < 0.001).

**Fig. 2:**
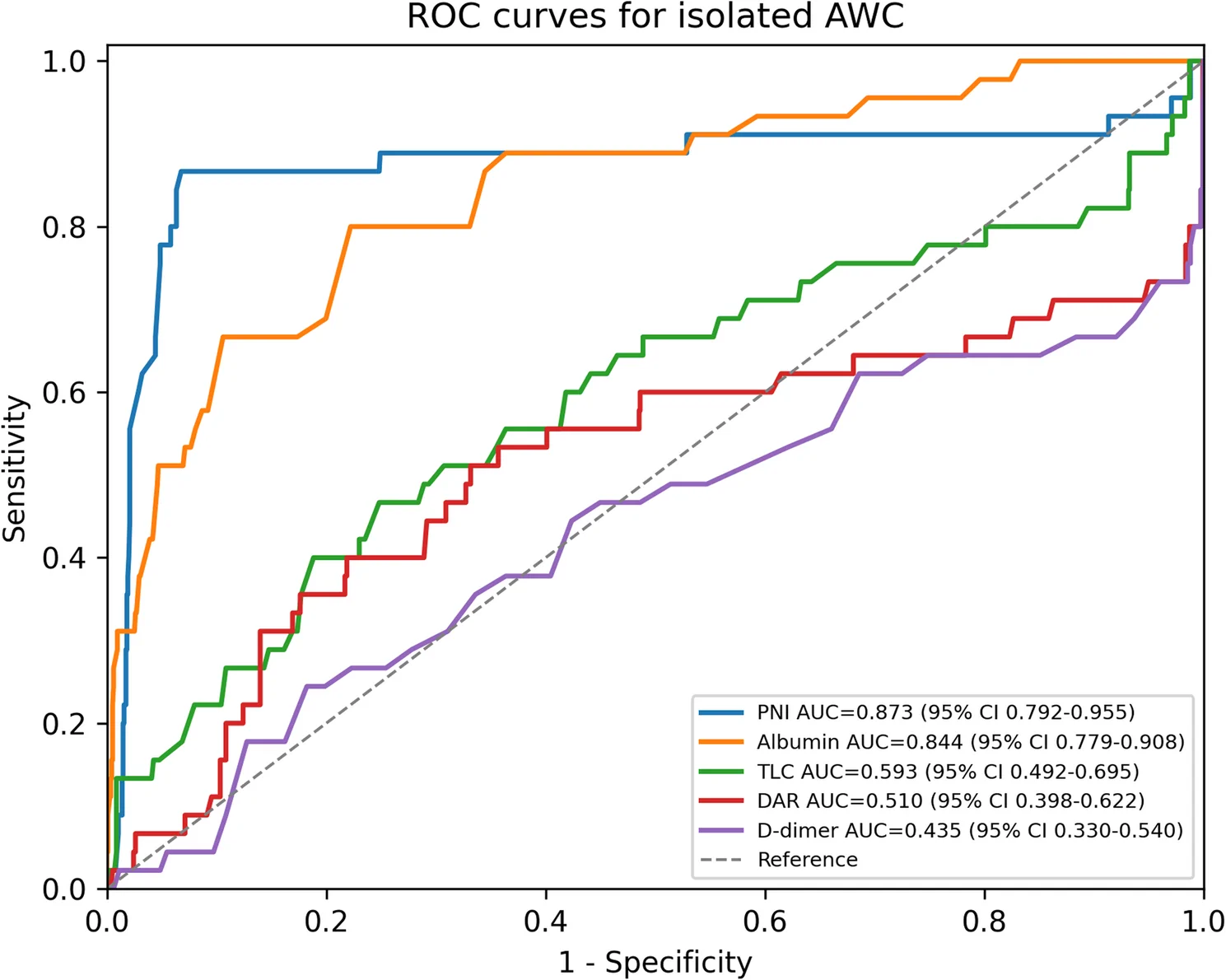
ROC curves of preoperative DAR, PNI, and component markers for discriminating isolated AWC. AUC values are shown with 95% confidence intervals

**Fig. 3:**
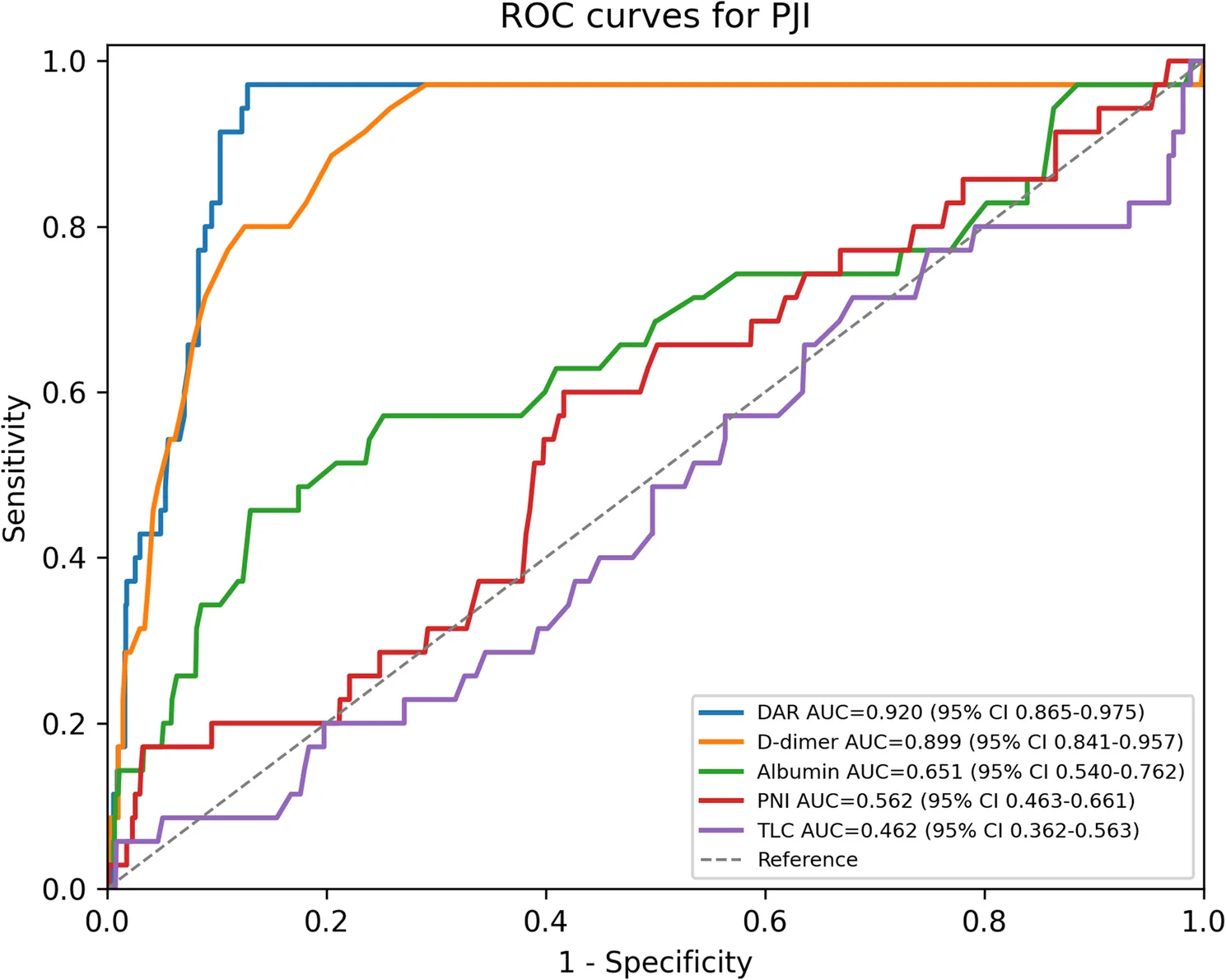
ROC curves of preoperative DAR, PNI, and component markers for discriminating PJI. AUC values are shown with 95% confidence intervals

For PJI, DAR showed the highest AUC (0.920; 95% CI, 0.865–0.975), with an exploratory cutoff of 11.19 (sensitivity, 97.1%; specificity, 87.2%; PPV, 18.6%; NPV, 99.9%). D-dimer also showed high discrimination (AUC, 0.899; 95% CI, 0.841–0.957), but DeLong testing indicated that the AUC of DAR was significantly higher than that of D-dimer (*P* = 0.014). Despite the high AUC and NPV values, PPV values were low because of the low event prevalence (Table 5; Figs. 2 and 3).

**Table 5:** ROC analyses of preoperative markers for isolated AWC and PJI

Outcome	Marker	AUC (95% CI)	Cutoff	Sensitivity	Specificity	PPV	NPV
Isolated AWC	DAR	0.510 (0.398–0.622)	≥ 10.46	40.0%	78.1%	6.7%	97.1%
Isolated AWC	PNI	0.873 (0.792–0.955)	≤ 49.95	86.7%	93.2%	33.3%	99.4%
Isolated AWC	D-dimer	0.435 (0.330–0.540)	≥ 490	24.4%	81.8%	5.0%	96.5%
Isolated AWC	Albumin	0.844 (0.779–0.908)	≤ 43.00	80.0%	77.8%	12.3%	99.0%
Isolated AWC	TLC	0.593 (0.492–0.695)	≤ 1.52	46.7%	75.2%	6.8%	97.3%
Isolated AWC	CRP	0.586 (0.503–0.669)	≥ 1.56	53.3%	71.4%	6.8%	97.5%
Isolated AWC	ESR	0.529 (0.453–0.606)	≥ 7	84.4%	30.2%	4.5%	98.0%
PJI	DAR	0.920 (0.865–0.975)	≥ 11.19	97.1%	87.2%	18.6%	99.9%
PJI	PNI	0.562 (0.463–0.661)	≤ 53.35	60.0%	58.3%	4.2%	98.0%
PJI	D-dimer	0.899 (0.841–0.957)	≥ 450	94.3%	74.2%	9.9%	99.8%
PJI	Albumin	0.651 (0.540–0.762)	≤ 42.20	45.7%	86.9%	9.5%	98.2%
PJI	TLC	0.462 (0.362–0.563)	≤ 1.01	5.7%	99.1%	16.7%	97.2%
PJI	CRP	0.546 (0.459–0.634)	≥ 0.50	60.0%	51.9%	3.6%	97.7%
PJI	ESR	0.541 (0.428–0.653)	≥ 13	45.7%	68.0%	4.1%	97.7%

Cutoffs were derived using the Youden index and should be interpreted as exploratory. DeLong tests: PNI vs. albumin for isolated AWC, *P* = 0.293; DAR vs. D-dimer for PJI, *P* = 0.014. Abbreviations: *AUC *area under the curve, *CI *confidence interval, *PPV *positive predictive value, *NPV *negative predictive value

### Internal validation, calibration, incremental value, and sensitivity analyses

In bootstrap internal validation, the optimism-corrected AUC was 0.873 for the univariable PNI-AWC analysis and 0.862 for the adjusted PNI-AWC model. For PJI, the optimism-corrected AUC was 0.919 for the univariable DAR-PJI analysis and 0.900 for the adjusted DAR-PJI model. The optimal PNI cutoff for AWC was stable in bootstrap resampling, with a median cutoff of 49.95 and interquartile range of 49.90-49.95; the DAR cutoff for PJI was also stable, with a median cutoff of 11.19 and interquartile range of 11.19–11.22.

Calibration assessment showed low Brier scores for the main models (0.031 for the adjusted PNI-AWC model and 0.027 for the adjusted DAR-PJI model), but Hosmer-Lemeshow testing suggested imperfect calibration for these key models. Decision-curve analysis suggested positive net benefit over selected clinically relevant threshold probabilities; however, these analyses were exploratory because of the low event rates and absence of external validation.

When standard laboratory markers (WBC, CRP, and ESR) were added to clinical covariates, the addition of PNI substantially increased the AUC for isolated AWC from 0.599 to 0.873 (likelihood-ratio *P* < 0.001; DeLong *P* < 0.001). Similarly, the addition of DAR increased the AUC for PJI from 0.593 to 0.924 (likelihood-ratio *P* < 0.001; DeLong *P* < 0.001). After clinical and standard laboratory adjustment, the AUC of the DAR model remained significantly higher than that of the corresponding D-dimer model for PJI (0.924 vs. 0.904; DeLong *P* = 0.007).

Sensitivity analyses that additionally included smoking status, inflammatory markers, operative time, estimated blood loss, and drainage volume yielded materially unchanged marker associations. Because the EPV was low in these extended models, these results were interpreted as exploratory rather than definitive (Table 6).

**Table 6:** Summary of internal validation, calibration, EPV, and sensitivity analyses

Analysis	Outcome/model	Key result
EPV assessment	Main AWC model	45 events / 6 predictors; EPV = 7.5
EPV assessment	Main PJI model	35 events / 6 predictors; EPV = 5.8
Bootstrap validation	PNI-AWC adjusted model	Apparent AUC 0.872; optimism-corrected AUC 0.862
Bootstrap validation	DAR-PJI adjusted model	Apparent AUC 0.922; optimism-corrected AUC 0.900
Calibration	PNI-AWC adjusted model	Brier score 0.031; Hosmer-Lemeshow *P* < 0.001
Calibration	DAR-PJI adjusted model	Brier score 0.027; Hosmer-Lemeshow *P* = 0.014
Sensitivity: inflammation + smoking	PNI-AWC	Adjusted OR 0.613 (95% CI, 0.542–0.693); *P* < 0.001
Sensitivity: surgical variables	PNI-AWC	Adjusted OR 0.613 (95% CI, 0.542–0.693); *P* < 0.001
Sensitivity: inflammation + smoking	DAR-PJI	Adjusted OR 1.448 (95% CI, 1.321–1.588); *P* < 0.001
Sensitivity: surgical variables	DAR-PJI	Adjusted OR 1.458 (95% CI, 1.330–1.597); *P* < 0.001

Extended sensitivity models had low EPV and are therefore exploratory. Abbreviations: *EPV *events per variable, *OR *odds ratio, *CI *confidence interval

### Descriptive distributions of PJI timing and AWC subtypes

Among the 35 PJI cases, 9 were diagnosed within 90 days after TKA and were classified as acute postoperative PJI. Twenty-six cases were diagnosed between 91 days and 24 months after surgery; of these, 25 were classified as delayed non-hematogenous PJI, and 1 was classified as presumed hematogenous PJI related to oral Candida albicans. Eight of the 9 acute postoperative PJI cases had a concomitant wound problem; the documented wound findings included persistent incisional drainage in 5 patients, hematoma in 2 patients, and wound dehiscence in 1 patient. The wound-dehiscence case was culture-negative and was managed with DAIR followed by two-stage revision. Overall, cultures identified Staphylococcus aureus in 10 PJI cases, Escherichia coli in 11 cases, and Candida albicans in 1 presumed hematogenous case, whereas 13 cases were culture-negative. Acute postoperative PJI cases were managed with DAIR-based treatment, with one patient subsequently undergoing two-stage revision; all delayed-period PJI cases, including the presumed hematogenous case, underwent two-stage revision (Table 7).

**Table 7:** Descriptive distribution of PJI timing, culture results, and treatment

PJI category / feature	Definition or description	*n*
Acute postoperative PJI	PJI diagnosed within 90 days after index TKA	9
Delayed-period PJI by timing	PJI diagnosed between 91 days and 24 months after surgery	26
Delayed non-hematogenous PJI	Delayed-period PJI without definite hematogenous evidence	25
Presumed hematogenous PJI	Delayed-period PJI related to oral Candida albicans	1
Concurrent AWC among acute postoperative PJI	Documented wound problem among acute postoperative PJI cases	8/9
Subtype of concurrent wound findings among acute postoperative PJI	Persistent incisional drainage / hematoma / wound dehiscence	5 / 2 / 1
Staphylococcus aureus culture	Synovial fluid or intraoperative culture	10
Escherichia coli culture	Synovial fluid or intraoperative culture	11
Candida albicans culture/source	Presumed oral-source hematogenous infection	1
Culture-negative PJI	No organism isolated in available cultures	13
Treatment of acute postoperative PJI	DAIR-based treatment; one culture-negative wound-dehiscence case subsequently underwent two-stage revision	9; 1 subsequent two-stage revision
Treatment of delayed-period PJI	Two-stage revision, including the presumed hematogenous case	26

TKA, total knee arthroplasty; PJI, periprosthetic joint infection; AWC, aseptic wound complications; DAIR, debridement, antibiotics, and implant retention. PJI subtype classification was descriptive and did not replace the 2018 ICM diagnostic criteria. The presumed hematogenous case occurred during the delayed-period time window

Among the 45 isolated AWC cases, delayed wound healing was the most common subtype (24 cases), followed by persistent incisional drainage (8 cases), wound dehiscence (8 cases), and hematoma (5 cases). Persistent drainage was managed with dressing changes. Among hematoma cases, 4 were managed with dressing care and 1 underwent debridement and secondary suturing. All 8 wound dehiscence cases underwent debridement and secondary suturing. Among delayed-healing cases, 20 were managed with delayed suture removal and continued dressing changes, whereas 4 underwent debridement and secondary suturing (Table 8).

**Table 8:** Subtype distribution and management of isolated AWC

AWC subtype	*n*	Main management
Persistent incisional drainage	8	Dressing changes/local wound care
Hematoma	5	4 dressing care; 1 debridement and secondary suturing
Wound dehiscence	8	Debridement and secondary suturing
Delayed wound healing	24	20 delayed suture removal and continued dressing changes; 4 debridement and secondary suturing
Total isolated AWC	45	Patients with concomitant PJI were assigned to the PJI group only

*AWC *aseptic wound complications, *PJI *periprosthetic joint infection.

## Discussion

This study investigated the associations of preoperative DAR and PNI with two different types of adverse outcomes after primary unilateral TKA. Overall, preoperative PNI was independently associated with isolated AWC, whereas preoperative DAR was independently associated with PJI. ROC analysis, DeLong comparison, bootstrap internal validation, and sensitivity analyses provided convergent but exploratory support for these associations. Importantly, these findings should not be interpreted as definitive evidence of stable prediction or as support for stand-alone clinical decision thresholds.

The association between lower PNI and isolated AWC is consistent with previous evidence linking malnutrition, hypoalbuminemia, and low PNI to postoperative wound-related complications after joint arthroplasty [6–9]. Hanada et al. reported that lower preoperative PNI was associated with aseptic wound complications after TKA, with the AWC group showing low PNI values in a similar range to the cutoff observed in the present study [6]. Our optimal cutoff of 49.95 was derived internally and was close to the mean PNI value of the AWC group. Although bootstrap resampling suggested internal cutoff stability, this threshold requires external validation before clinical use.

From a biological perspective, PNI incorporates albumin and TLC and therefore may reflect combined nutritional and immune reserve. Albumin is related to colloid osmotic pressure, tissue repair, collagen synthesis, and wound-microenvironment stability, whereas lymphocytes participate in immune regulation and tissue-repair processes. However, because this study is observational, these mechanistic explanations should be considered biologically plausible interpretations rather than causal evidence.

In contrast, DAR showed the strongest association with prior studies, including an early serum D-dimer diagnostic study and subsequent systematic reviews, have reported potential diagnostic value of D-dimer for PJI, although between-study heterogeneity is substantia [15–18, 24]. In our analysis, DAR had a numerically and statistically higher AUC than D-dimer alone for PJI. After adjustment for clinical variables and standard inflammatory markers, DAR also showed incremental discrimination beyond WBC, CRP, and ESR. This suggests that DAR may capture information not fully represented by conventional inflammatory markers, although this interpretation remains exploratory.

The interpretation of DAR and PNI must consider their shared albumin component. Low albumin contributes to both a higher DAR and a lower PNI, but the two indices combine albumin with different biological dimensions: D-dimer in DAR and TLC in PNI. Thus, the differential associations observed here should not be taken to indicate fully independent biological pathways. Rather, they may indicate that albumin-related nutritional or inflammatory vulnerability is expressed differently when combined with coagulation-fibrinolytic activation versus lymphocyte-based immune reserve.

The separation of isolated AWC and PJI was intended to address outcome heterogeneity. AWC occurs early after surgery and is more directly influenced by local soft-tissue conditions and wound-repair capacity, whereas PJI may involve longer-term host-pathogen interactions and systemic inflammatory-coagulation responses. Nevertheless, early wound complications and infection exist on a related clinical spectrum. In this cohort, 8 of the 9 acute postoperative PJI cases had concomitant wound abnormalities, including 5 cases of persistent incisional drainage, 2 hematomas, and 1 wound dehiscence, supporting the clinical overlap between early wound disturbance and infection. In addition, one case diagnosed during the 91-day to 24-month window was classified as presumed hematogenous PJI related to oral Candida albicans, illustrating that PJI events captured during longer follow-up can have heterogeneous clinical routes. Because the number of cases within each subtype was small, subtype-specific regression analyses were not performed; the subtype data were therefore used descriptively to contextualize the primary findings.

The clinical utility of these markers should be interpreted cautiously. Although the AUCs and NPVs were high, PPVs were low because of the low event prevalence. Therefore, PNI and DAR should not be used as stand-alone screening or decision tools. Their potential value may lie in adjunctive risk stratification, identification of patients who merit closer follow-up, and recognition of patients with low predicted risk. Decision-curve analysis suggested potential net benefit in selected threshold ranges, but this finding requires validation in external cohorts.

This study has several limitations. First, it was a single-center retrospective study, which limits external validity and exposes the analysis to selection bias and information bias. Second, the number of outcome events was limited, especially for PJI. The EPV of the primary PJI model was below 10, and the extended models had even lower EPV; therefore, all expanded analyses should be interpreted as exploratory and hypothesis-generating. Third, although we added descriptive summaries of AWC subtypes and PJI timing/route, the sample sizes within these subgroups were small and did not support reliable subtype-specific multivariable modeling. In addition, several acute postoperative PJI cases had concomitant wound problems, reinforcing that AWC and PJI may overlap clinically and that early wound complications can occasionally represent or precede occult infection. Fourth, one presumed hematogenous PJI related to oral Candida albicans was identified, but the retrospective nature of the study still limited precise determination of infection route in every case. Fifth, the ROC-derived cutoffs were internally derived and showed imperfect calibration despite favorable apparent discrimination; they should therefore not be used as stand-alone clinical decision thresholds without external validation.

Several perioperative and patient-level factors were also incompletely captured. Although operative time, estimated blood loss, drainage volume, smoking status, WBC, CRP, and ESR were added to baseline comparisons and sensitivity analyses, other variables such as transfusion, implant subtype, deformity severity, extent of soft-tissue release, corticosteroid exposure, immunosuppressive therapy, and preoperative nutritional supplementation were not uniformly recorded. Post-discharge anticoagulation adherence was reinforced through education but not objectively quantified. These issues may have introduced residual confounding.

Our institutional pathways may also influence generalizability. All eligible patients underwent D-dimer testing and lower-extremity Doppler ultrasonography, and those with preoperative DVT were excluded. This strategy improved internal consistency for evaluating DAR but may have influenced the distribution of D-dimer and DAR and may limit applicability in institutions where routine Doppler screening is not performed. Similarly, chest CT availability reflected an institutional preoperative anesthetic and pulmonary risk assessment pathway for elderly patients rather than a universal TKA planning requirement. Current preoperative testing recommendations generally support selective testing based on patient characteristics, comorbidities, examination findings, and procedure invasiveness; therefore, our CT pathway should not be interpreted as a general recommendation for all arthroplasty candidates [20–22].

In summary, the present findings suggest that preoperative PNI may be more strongly associated with isolated AWC, whereas preoperative DAR may be more strongly associated with PJI in patients undergoing primary unilateral TKA. These observations support the concept that different postoperative complications may correspond to different host-status patterns. However, given the retrospective single-center design, low event numbers, low PPVs, imperfect calibration, and absence of external validation, the findings should be regarded as exploratory and hypothesis-generating. Larger multicenter prospective studies are needed to validate optimal cutoffs, external reproducibility, calibration, and incremental value beyond existing clinical risk models.

## Conclusions

Among patients undergoing primary unilateral TKA, preoperative PNI was independently associated with isolated AWC, whereas preoperative DAR was independently associated with PJI. In this single-center retrospective cohort, PNI for isolated AWC and DAR for PJI showed good apparent within-sample discrimination, but their low positive predictive values, imperfect calibration, limited number of events, and lack of external validation require cautious interpretation. Larger multicenter prospective studies are needed before these internally derived cutoffs can be used as clinical decision thresholds.

## Data Availability

The datasets generated and analyzed during the current study are available from the corresponding author on reasonable request.
